# Cellulose membrane as a biomaterial: from hydrolysis to depolymerization with electron beam

**DOI:** 10.1186/s40824-016-0065-3

**Published:** 2016-07-14

**Authors:** Mi Young Eo, Huan Fan, Yun Ju Cho, Soung Min Kim, Suk Keun Lee

**Affiliations:** Department of Oral and Maxillofacial Surgery, Dental Research Institute, School of Dentistry, Seoul National University, 101 Daehak-ro, Jongno-gu, Seoul, 110-768 South Korea; Department of Oral Pathology, College of Dentistry, Gangneung-Wonju National University, Gangneung, 123 Chibyon-dong, Gangneung, 210-702 South Korea

**Keywords:** Cellulose binding domain, Cellulose crosslinking protein, Cellulose membrane (CM), Depolymerization, Electron beam (E-beam) irradiation

## Abstract

The cellulose membrane (CM) is a major component of plant cell walls and is both a chemically and mechanically stable synthetic polymer with many applications for use in tissue engineering. However, due to its dissolution difficulty, there are no known physiologically relevant or pharmaceutically clinical applications for this polymer. Thus, research is underway on controlled and adjusted forms of cellulose depolymerization.

To advance the study of applying CM for tissue engineering, we have suggested new possibilities for electron beam (E-beam) treatment of CM. Treatment of CM with an E-beam can modify physical, chemical, molecular and biological properties, so it can be studied continuously to improve its usefulness and to enhance value.

We review clinical applications of CM, cellulose binding domains, cellulose crosslinking proteins, conventional hydrolysis of cellulose, and depolymerization with radiation and focus our experiences with depolymerization of E-beam irradiated CM in this article.

## Background

Electron beam irradiation (EBI), also known as electron beam (E-beam) processing, is a process that uses electrons, usually of high energy, to treat objects for purposes such as sterilization and crosslinking of polymers. E-beams have been used in many types of research, technology, and medical therapy fields and used in electron microscopes for the ultramicroscopic analysis of materials as well as to produce images on television screens [1–4].

Cellulose is a major component of plant cell walls and is the most abundant macromolecule on earth. It is also an inexpensive and abundant synthetic polymer with routine applications in its powdered form as a tablet binder or filler. It is chemically and mechanical stable as well as completely insoluble under physiological conditions, which makes the cellulose membrane (CM) an ideal candidate for medical modifications and for tissue engineering uses. However, due to its dissolution difficulties, there are no convenient systems known for using cellulose in physiologically relevant or pharmaceutically clinical applications. Cellulose depolymerization in controlled and adjusted forms is of interest to many researchers [5–8].

During our recent investigations of the effects of EBI on maxillofacial reconstructive polymer materials, we have tried to identify gross and elemental changes in E-beam irradiated CMs [9–11]. E-beam treatment involves accelerating a beam of electrons to near the speed of light and by utilizing an oscillating magnetic field, sweeping the electrons back and forth across the polymers. It can be thought to modify physical, chemical, molecular and biological properties. To advance the study of applying E-beam irradiated CM for tissue engineering approaches, here, we suggest new possibilities for EBI treatment of CM.

### Clinical applications of CM

**Fig. 1:**
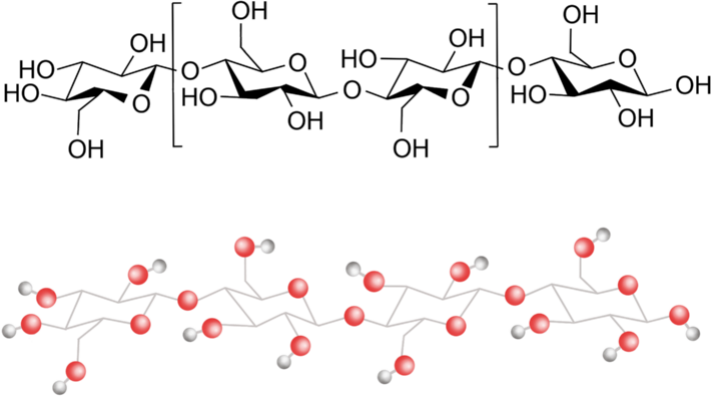
Schematic drawing of the basic structure of natural cellulose having a linear chain with β 1–4 linked D-glucose units

Cellulose is a naturally occurring polymer composed of repeating anhydroglucose units linked to gather by β 1–4 glycosidic linkages (Fig. 1). It is an organic polysaccharide compound with the formula (C_6_H_10_O_5_)_n_ consisting of a linear chain of several hundred to many thousand β 1–4 linked D-glucose units [5, 8, 10]. Based on the location of hydrogen bonds between and within strands of units, different crystalline structures of cellulose are known (cellulose I to IV). Two major allomorphs of cellulose consisting of a microfibrillar crystalline array of linear β1,4-glucan chains are found in nature; all naturally occurring cellulose allomorphs are oriented parallel to one another with the same polarity [12, 13]. Natural cellulose is cellulose I with structure Iα is produced mainly by bacteria and algae, while that with structure Iβ is the main composition of higher plants. The extended chain conformation of cellulose I allows the formation of microfibrils with extraordinary mechanical strength. Cellulose II is formed from cellulose I through chemical treatments such as mercerization that alter the crystal structure. The cellulose II allomorph is also produced by a few organisms in nature. The conversion of cellulose I to cellulose II is irreversible, suggesting that cellulose I is metastable and cellulose II is stable [14–16].

**Fig. 2:**
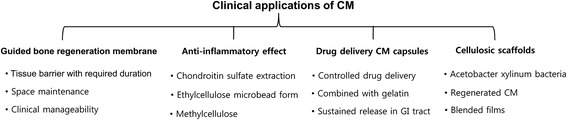
Summarized category of clinical applications of cellulose membrane

Cellulose polymers are known to have a good biocompatibility and wound healing characteristics like other natural polymers including alginates and chitosan. Much incorporative trial of such natural polymers in synthetic combinations can produce biomaterials with features of synthetic polymers and specific biocompatible and wound healing characteristics of natural polymers [17]. Thus, the application of natural polymers for medical or environmental purposes necessitates the use of these polymers in crosslinking hydrogels [18] or the chain scissioning with exposure to high energy radiation [14, 19]. We reviewed and summarized clinical applications of CM (Fig. 2).

### Guided bone regeneration membrane

In the clinical medical and dental fields, a variety of non-resorbable and absorbable barrier membranes for bony augmentation were used as the basis of guided bone regeneration (GBR). GBR was introduced to correct bony deficiency and has shown good clinical results over the last several years. The basic concept of GBR evolved from guided tissue regeneration; it is used to compartmentalize new osteogenesis using barrier membranes by protecting the blood clot, creating space, and excluding soft tissue cell proliferation [20, 21]. Several membranes, from non-resorbable expanded polytetrafluoroethylene membrane to absorbable membrane, including polyglactin 910 (coated Vicryl®), collagen, calcium phosphate or other intact connective tissue, have been shown to have specific characteristics necessary to manufacture GBR, such as material biocompatibility, stability over the required duration of barrier functions, space maintenance, exclusion of undesired cell ingrowth, and ease of use [9, 22].

We previously demonstrated that CM can be successfully used as a GBR membrane in combination with particulate bone grafting and that the peculiar characteristics of E-beam irradiated CM are also useful for space maintenance and biocompatibility [2, 5, 10, 11]. Five surgical factors are required to achieve predictable results with GBR procedures: 1) use of an appropriate membrane, 2) achievement of primary soft tissue healing, 3) creation and maintenance of a membrane-protected space, 4) close adaptation and stabilization of the membrane to the surrounding bone, and 5) a sufficiently long healing period. Other prerequisites for ideal barrier membranes are known including biocompatibility, cell occlusivity, tissue integration, space-making effect, and clinical manageability. E-beam irradiated CM fulfills one important surgical limitations faced with non-resorbable CM, but it has appropriate results in above all surgical factors and prerequisites. E-beam irradiated CM does not need a second surgical procedure for its removal due to its biocompatible longevity. The second surgery increases the risk of loss of regenerated bone on the flap reflection [1, 11].

### Anti-inflammatory effect of CM

Several purified molecules and included composition from CM have been shown to have anti-inflammatory and anti-cancer effects. Chondroitin sulfate (CS) extracted from the *Styela clava* tunic can significantly inhibit NF-kB driven expression of vascular cell adhesion molecule-1 and inducible nitric oxide synthase by blocking Akt signaling in JB6 cells [23]. CS is a naturally present glycosaminoglycan in the extracellular matrix of articular cartilage and is also known to have anti-inflammation and anti-cancer effects. Hyaluronic acid-carboxymethylcellulose membranes can be applied topically for postoperative scar tissue reduction; they decrease perineural scar formation and adhesion after sciatic nerve repair in rats and are also effective in promoting peripheral nerve regeneration at the repair site [24]. 5-Fluorouracil loaded calcium-zinc-gellan and calcium-zinc-gellan-ethylcellulose microbeads are also useful for sustained drug release, with a formulation ratio of drug:gellan:ethylcellulose at 2.5:7.5:1. Sustained drug release activity was found to provide more effective anti-cancer activity [25].

Methylcellulose (MC) has been used to control the gelation time of silk fibroin aqueous solution and in the design and tailoring of drug release of hydrogels by controlling the sol–gel transition [26]. During release procedures of donor or receiver fluid, regenerated CM remains chemically unchanged; its pore size remains constant, and no drugs are partitioned into the membrane [27]. CM is also recognized as a permeation enhancer. Improved permeation of diclofenac diethylamine using isopropyl myristate and isopropyl palmitate as permeation enhancers for fabrication of topical formulations was also observed when bacterial cellulose was used as the diffusion membrane [28]. More recently, the effect of the molecular weight of MC on gelation viscosity and strength of ophthalmic formulations was confirmed in increased drug release properties among different formulations [29].

### Drug delivery CM capsules

Due to the inert biologic and chemical characteristics of CM, it is considered as an ideal candidate for capsule-based controlled drug delivery.

Two piece hard shell variant telescoping capsules are rarely used for controlled drug delivery. The feasibility for controlled release using CM can be determined in a relatively short time with small quantities of bulk drug, especially when dealing with early drug candidates [30]. Moreover, since the capsule properties can be independently modulated without interacting with the core formulation, this dosage form is suitable for drug molecules that are difficult and expensive to obtain and for those sensitive to aqueous or organic environments and the elevated temperatures typically encountered during tablet coatings used for controlled release [31].

The gelatin capsule is the most common starting components, and gelatin dissolution times in the gastro-intestinal tract have been investigated to improve the feasibility of using gelatin capsules for delayed and extended drug delivery [32–34]. Recently, drug release performance from regenerated cellulose capsules was studied, and an additional coating over hard gelatin capsules to achieve controlled, delayed, and or sustained release from capsules was approved in spite of the presence of pepsin and pancreatin in the stomach and intestinal fluids [33, 35]. This cellulose capsule possessed the advantages of ease of use and portability. Capsules are a popular dosage form because they provide a smooth, slippery, easily swallowable, and tasteless shell for drug delivery; they are particularly beneficial for drugs with unpleasant tastes and odors [30, 31]. Thus, gelatin capsule made from regenerated cellulose are commonly used in the commercial fabrication market of two piece telescoping capsules used for controlled oral drug delivery.

### Cellulosic scaffolds

In addition to its role as a drug delivery system, cellulosic scaffolds can also be used for tissue engineering [6, 7] and other bio-medical applications. The process of cellulose generation and scaffold fabrication involves the use of *Acetobacter xylinum* bacteria in a glucose rich environment to produce a highly purified cellulose matrix with high degrees of swelling. *A. xylinum* is a representative bacteria in which the physiochemical properties of the cellulose matrix are controlled by changes in growth medium to obtain the desired functionality, and different kinds of cell seeding and tissue growth, as well as the addition of non-biological products, can easily be achieved by placing living cells in the same growth medium as the *A. xylinum* bacteria [36].

Regenerated CM is commonly used in protein separation and reverse osmosis processes such as diffusion induced phase separation precipitation of cellulose solutions in aqueous environments, thermal annealing in various organic non-solvents, or polymer consolidation (hornification processes) [37]. However, there is very little information on the properties of these scaffold membranes formed after diverse processes.

More recently, blended films have been prepared from native cotton linters and depolymerized cotton linters to alter regenerated CM properties such as water uptake, porosity, and tortuosity [38]. The changes in solute size hydrophobicity were achieved through E-beam irradiated regenerated CM [11, 17]. The structure and properties of these membranes could be controlled and modified depending on the energy, type, and E-beam dose [39].

### Cellulose binding domains and cellulose crosslinking proteins

**Fig. 3:**
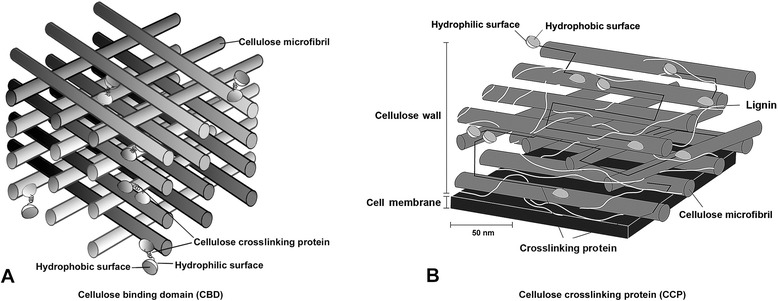
Schematic drawing of the cellulose binding domain (**a**) and cellulose crosslinking protein with cellulose microfibril and lignin (**b**) [confirmed permission from reference journal 11]

Cellulose binding domains (CBDs) are structurally and functionally independent; no catalytic modules nor essential elements are found in many cellulose or hemicelluloses degrading enzymes (Fig. 3) [11, 40, 41]. All CBDs have affinity for cellulose; they are divided into several families and do not have any hydrolytic activity [40]. Many organisms produce different CBDs, providing immense potential for applications of CBDs in the field of biotechnology. CBDs were recently used to facilitate protein immobilization on cellulose supports.

Cellulose is an ideal matrix for large-scale affinity purification procedures. This chemically inert matrix has excellent physical properties as well as low affinity for nonspecific protein binding [42]. CBDs can be removed from enzymes by proteolysis or by protein engineering, and CBDs can be applied in the modification of physical and chemical properties of composite materials and the development of modified materials with improved properties. It is available in a diverse range of forms and sizes, is pharmaceutically safe, and is relatively inexpensive. However, at the molecular level, the role of the CBD in the hydrolytic action of enzyme remains unclear.

The affinity of CBDs can be significantly improved by fusing two CBDs together using a linker to form a double CBD [43]. Most cellulose-degrading enzymes have a two-domain structure that consists of a catalytic domain and a CBD connected by a linker region. The linkage and the interactions of the two domains represent the function of these enzymes. Phenotypically, the removal of CBD from the enzyme results in decreased affinity and much reduced hydrolytic activity on crystalline cellulose.

### Classification and purifications of cellulose binding domains

Family I CBD can reversibly bind to cellulose, while Families II and III CBD display irreversible binding. When Family I CBD is used as an immobilizing tag, low-rate column leakage is often observed [44]. Fungal Family I CBD differs from bacterial Family II CBD in size and structure. Early experiments with the *Trichoderma reesei* cellobiohydrolase I (CBHI) CBD suggested that these CBDs bind irreversibly to cellulose columns [45]. Linder et al. [44] constructed a chimeric protein that was composed of CBD HII and CBHI from *Trichoderma reesei* and a single-chain antibody. In this procedure, measurement of the reversibility and exchange rates was very difficult for its sensitization of tritium labeled proteins [44].

Brun et al. [46] proposed a novel two-phase separation system to purify proteins from aqueous solutions utilizing Family IV CBD that bind to water-soluble cellulosic materials such as hydroxyethylcellulose [46]. The system was composed of phase-forming polysaccharide polymers to which CBD can bind and a phase-inducing agent such as polyethylene glycol. The solution containing the CBD-fused peptide or protein was mixed with the phase-forming oligosaccharide followed by the addition of the phase-inducing agent. The two phases were then separated, and the target protein was purified. This system can be very effective for the separation of proteins from fermentation broths as well as from other aqueous solutions. Most work on CBD-mediated protein immobilization has been carried out using family II CBDs, especially those from *Cellulomonas fimi* [47]. The leaking of immobilized proteins has been studied, but usually none is detected, thus leakage has not been regarded as a problem [48]. This has led to the hypothesis that the interaction of these CBDs with cellulose is irreversible and the utilization of CBDs has advantages for the many production of CBD fusion proteins in plants by use of E-beam.

### Targeting of cellulose binding domains

From the many technical applications for CBD binding, the most common and first commercial application was the use of CBDs in fusion proteins as tags for affinity purification or immobilization. Since CBDs spontaneously adsorb to cellulose from almost any solution, very little or no pretreatment of the samples is required prior to immobilization. Thus, CBDs offer many industrially attractive uses.

Cellulose is a major constituent of many commercial products; therefore, targeting functional molecules to cellulose-containing materials can be mediated by CBDs. The commercial potential of CBD in this context was first realized for denim stonewashing [49]. With the introduction of recombinant enzyme technology, the strong affinity between cellulose and CBD was utilized for enzyme targeting to garments. This development eventually evolved into an alternative process that completely replaced the traditional stones [49].

The strong affinity between cellulose micrifibrils and CBDs is used in many applications associated with the textile industry. Numerous laundry powders contain recombinant enzymes that do not possess a native affinity for the cellulosic fabrics. The performance of these enzymes under conventional washing conditions can be improved by increasing their affinity to the textile substrate [50], and this can be achieved by fusion to CBDs. Additional substances can also be targeted to cellulosic fabrics. For example, fragrance-bearing particles conjugated to CBD can be added to laundry powder, which reduces the amount of fragrance needed in the product.

Threads are exposed to considerable mechanical strain during the weaving process. To prevent tearing, the threads are reinforced by gelatinous substances by a process called ‘sizing’. The most popular material used for sizing is starch, but cellulose derivatives such as carboxymethylcellulose (CMC), hydroxyethylcellulose, hydroxypropylcellulose, and MC are also employed. A contradictory effect of the sizing agents is that fabrics are not able to absorb water-based finishing agents, such as dyes. To improve the enzymatic ‘desizing’ process, target enzymes can be fused to CBD, thus increasing the affinity of the enzymes for the cellulosic fabrics [50, 51].

Antimicrobial agents can be targeted to polysaccharide materials. Emerson et al. [52] proposed the targeting of aromatic aldehydes or alcohols to cellulose-containing materials. Aromatic aldehydes and alcohols, including benzaldehyde, acetaldehyde cinnamaldehyde, piperonal, and vanillin, are known to be effective disinfectants for bacteria, fungi, and viruses and are nontoxic to humans and animals. Targeting can be attained with the assistance of CBD and may be useful for directly impregnating surfaces such as paper or wood.

Another interesting application of CBD is in oral care products. Polysaccharides such as fructan and glucan that are present in the oral cavity are known to be involved in the formation of dental plaque; these sugars can be degraded by CBD fused to enzymes. Fuglsang and Tsuchiya [53] concluded that CBD on its own or in combination with other ingredients removes existing plaque or prevent its formation when used in conventional oral hygiene.

### Cellulose crosslinking proteins (CCPs)

Cellulose crosslinking proteins (CCPs) are found as essential and efficient cross-linkers between hydrophilic surfaces of different CBD, which enables each cellulose microfibril to crosslink in the final cellulosic materials (Fig. 3). In more recent biotechnology, CCPs with CBD linking has been used to modify polysaccharide materials both in vivo and in vitro. The potential applications of CCPs technology range from modulating the architecture of individual cells to modifying entire organisms in tissue engineering [42, 43].

Targeting and applying CCP to cellulose fibers may be of potential use [40, 41] in the paper recycling industry. CBDs exert nonhydrolytic fiber disruption on cellulose-containing materials. Application of a single CBD molecule to paper can improve its mechanical properties but to a lesser extent when compared to CCP. In addition, papers treated with CCP become more hydrophobic and demonstrate water repelling properties. At high CCP concentrations, most binding sites on cellulose are occupied by single CBD moieties; consequently, the second unbound CBD moiety of CCP exposes a hydrophobic surface and, in this manner, increases surface hydrophobicity. At optimum CCP concentrations, all binding sites in CCP are attached to the cellulosic surface, which results in improved mechanical properties [42, 43]. It has been demonstrated that the application of CBD on secondary fibers, such as old paperboard containers, results in increased tensile and burst indexes as well as improved pulp drainage [42].

### Our experiences with E-beam irradiated CM

To create a bone regeneration membrane, we developed CM from native sea squirt skin, called non-native tunicate or *Styela clava*, which inhabits sea facing bays and harbors. Previously, we determined that a CM was success sful for use as a GBR barrier in combination with particulate bone grafting [5, 9, 10]. Additionally, the peculiar characteristics of E-beam irradiated CM were found to be useful in space maintenance and biocompatibility [11]. E-beam irradiated CM overcomes some of the limitations of non-resorbable CM such as the need for a second surgical procedure for its removal.

**Fig. 4:**
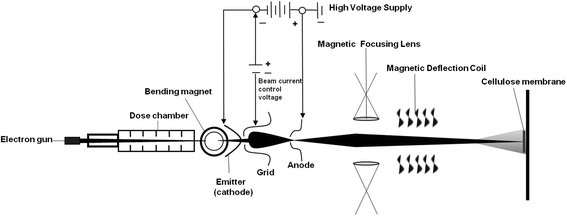
Schematic drawing of E-beam irradiation of CM, showing the basic linear accelerator including the electron gun, cathodic emitter, grid, anode, magnetic focusing lens and magnetic deflection coil

**Fig. 5:**
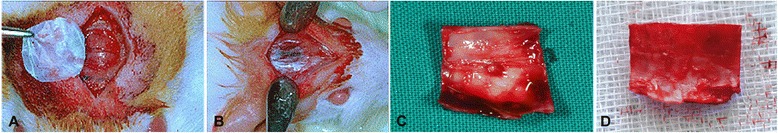
Photomicrographs of the standardized transosseous circular 8.0 mm sized calvarial defect with E-beam irradiated CM (**a**), the covered defect with different conditioned E-beam irradiated CM (**b**), and the inner side of the frontal bone including the perforated area and periosteal membrane at 12 weeks (**c**) and 24 weeks (**d**)

To evaluate the effect and potential of E-beam irradiated CM, we used a 1.0 MeV linear accelerator or a 2.0 MeV superconductive linear accelerator (power 20–300 kW, pressure 115 kPa, temperature −30–120 °C, sensor sensitivity 0.1–1.2 mV/kPa, generating power sensitivity 44.75 mV/kPa, supply voltage 5 ± 0.25 V) with different irradiation doses (1, 10, 30, and 120 kGy; Fig. 4). Structural changes in CM were studied in vitro by elementary and amino acid analysis, elementary analysis using field emission scanning electron microscope, electron spectroscopy for chemical analysis (ESCA), attenuated total reflection infrared analysis, and scanning electron microscopy (SEM). And we compared changes under different conditioned E-beam irradiated CM in an in vivo animal study, which were applied on standardized transosseous circular 8.0 mm sized calvarial defect 8-weeks-old, Sprague–Dawley male rats (Fig. 5) under the approvement of Seoul National University Institutional Animal Care and Use Committee (SNU-120801-3-3).

E-beam treatment involves accelerating a beam of electrons to near the speed of light and, by using an oscillating magnetic field, sweeping the electrons back and forth across the CM [1, 2]. From our analysis, CM has a pure carbohydrate polymer structure consisting of a rigid outer surface and a delicate inner surface; this structure lends itself to development as a tissue regenerative barrier membrane [5, 10]. Very small amounts of peptide fragments derived from E-beam treated CM, such as CCP, which is a kind of anchoring protein composed of glycocalyx, could be lost its own structure [11].

**Fig. 6:**
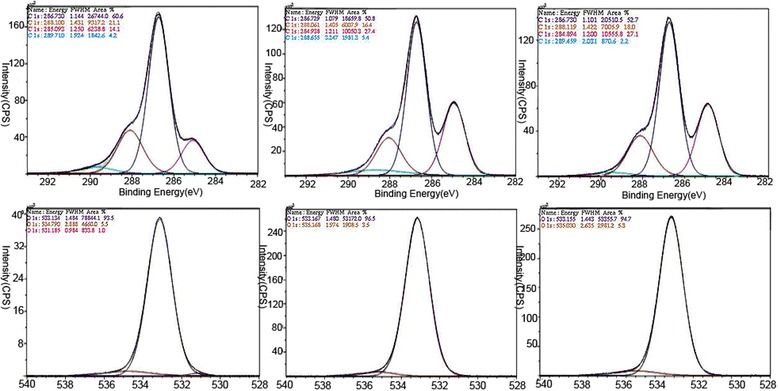
A high-resolution C1s ESCA spectrum showing hydrocarbon (C-C, C-H) bonding at the 284.9 eV peak, hydroxyl carbon (C-O-H) bonding at the 286.7 eV peak, ether carbon (O-C-O) bonding at the 288.4 eV peak, and ester carbon (O-C = O) bondings at the 289.6 eV peak. No E-beam (upper left), inner (upper middle), and outer (upper right) surface of 2 MeV - 0.24 mA - 120 kGy irradiated CM. A high-resolution O1s ESCA spectrum showing hydroxyl oxygen (−C-O-H), ether oxygens (−C-O-C-), and ester oxygen (−COO). No E-beam (lower left), inner (lower middle) and outer (lower right) surface of 2 MeV - 0.24 mA - 120 kGy irradiated CM [confirmed permission from reference journal 11]

Cellulose is a carbohydrate polymer composed of carbon, hydrogen, and β-glucose and is the main composition of plant cell walls. Glucose is the major constructive carbohydrate in 95 % of cellulose, while fucose, arabinose, and mannose make up 1–2 % each. There are more C-O bonds than C-C bonds, and several related results showed that depolymerization of cellulose microfibrils forms microtubules. The C = O functional group is also present in both the organic and non-organic synthetic materials, and surface chemical bonding energy of each carbon and oxygen has been confirmed through C1s and O1s spectra in ESCA (Fig. 6) [11].

**Fig. 7:**
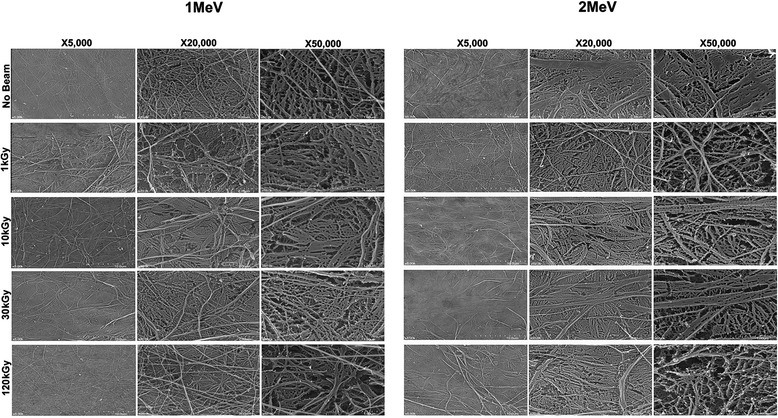
SEM findings of the inner surface of 1 MeV and 2 MeV E-beam irradiated CM according to 1, 10, 30 and 120 kGy doses at × 5.0 k, × 20.0 k, and × 50.0 k magnifications

Scissioning of long carbohydrate polymers can be observed under SEM (Fig. 7), and uniform turgor pressure to maintain the directionality of the CM can be changed to be elongated after EBI. Arrangements of microtubules can also be changed indirectly, so cellulose and enzyme complexes are able to migrate in the plasma membrane. Tensile strength of CM can be changed because polysaccharide cross-linking is able to lose its resistance to compression and modify its physical, chemical, molecular and biological properties. CCP was lost after EBI, leading to detachment of the strong crosslinking binding of each fibril [11, 22].

**Fig. 8:**
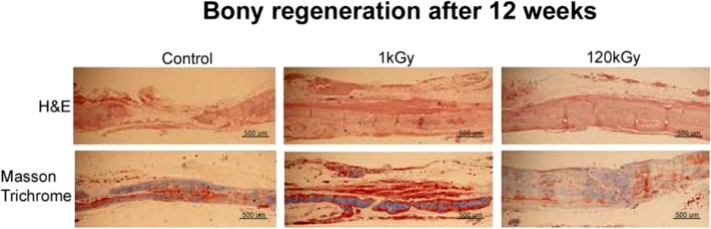
H&E and masson trichrome staining of coronal sections of the regenerated frontal bone underneath each different conditioned CM (×40 magnification)

**Fig. 9:**
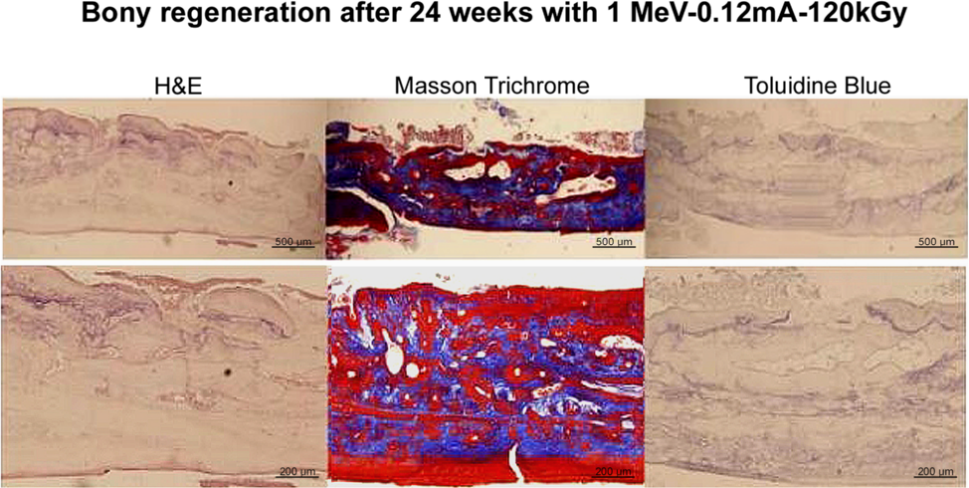
H&E, masson trichrome, and toluidine blue staining of coronal sections of the regenerated frontal bone underneath 1 MeV-120 kGy E-beam irradiated CM (×40 and × 100 magnifications)

In light microscopic observations of the regenerated calvarial defects after 12 and 24 weeks, new bone formation was histologically more active in the 12 weeks specimens with EBI than in the sham or control groups (Fig. 8). In the 24 weeks specimens, some part of CM was partially degraded, as observed under H&E, Masson Trichrome, and toluidine blue stains (Fig. 9). The potential of E-beam irradiated CM polymers as thin um thickness medical membranes for guided bone regeneration and the possibilities of clinical application of E-beam irradiated CM as biodegradable or resorbable membranes was confirmed.

### Depolymerization of CM

For the chronological understandings of cellulose depolymerization, we separated the subheadings from the hydrolysis of cellulose to the radiation induced and E-beam irradiation.

### Degradation or hydrolysis of cellulose

The natural polymer form of cellulose is composed of repeating anhydroglucose units linked together by β 1–4 glycosidic linkages. The native regular patterns of crystalline cellulose in electron diffraction are cellulose microfibrils. Cellulose could be assembled abiotically by many kinds of enzymatic polymerization. For example, cellulose from cotton linter sheets can be depolymerized according to the mineral acid hydrolysis method [54, 55]. Briefly, cotton linter sheets were shredded into 1 cm × 1 cm pieces, and sections weighing approximately 50 g were placed in a 1 l flask filled with 500 ml of 2 N hydrochloric acid and agitated using a magnetic stir bar at 40 °C in a thermostatic water bath for up to 72 h. At predetermined time intervals, approximately 1 to 5 g samples were removed from the flask and rinsed under running water for 2 h, followed by washing in acetone. Finally, the sections were placed in an oven at 105 °C for 24 h. At the end of 72 h, the remaining cellulose slurry was rinsed and dehydrated by the same method [54].

Until the early 21st century, only two major allomorphs of natural cellulose biopolymer were known: cellulose I and II. Cellulose I and II both consist of a microfibrillar crystalline array of linear β 1,4-glucan chains, all of which are oriented parallel to one another with the same polarity. The extended chain conformation of cellulose I allows for the formation of microfibrils with extraordinary mechanical strength. Cellulose II and its various allomorph were recently shown to be formed from cellulose I by altering the crystalline structures, for example by mercerization, recrystallization, or polymerization [55, 56].

Abiogenic synthesis of various cellulose allomorphs has been produced by a few organisms in nature. Dissolution or degradation of cellulose microfibrils also occurs in nature. Brown-rot fungi in decaying wood is a representative natural organism that rapidly depolymerizes cellulose during the early stages of wood decay by producing endo-1–4 β-glucanases [55]. In wood decayed by brown-rot fungi, the hemicelluloses fraction is virtually absent, and the degree of cellulose depolymerization is abruptly reduced. Crystalline cellulose can be also degraded by a synergistic action between endo- and exo-glucanases in the case of white-rot fungi such as *Trichoderma reesei*, which is an important industrially used microorganism with cellulose production ability, and *Sporotrichum pulverulentum*, which has abundant cellulases induced by cellobiose dehydrogenase and repressed by small amounts of glucose [55, 57]. These white-rot fungi depolymerize cellulose more slowly and utilize the degradation products simultaneously, while brown-rot fungi depolymerize cellulose rapidly during early stages of wood decay and produce abundant amounts of cellulases with glucose as the only carbon source [57, 58].

Brown-rot fungi is known to lack the synergistic endo- and exo-glucanase cooperation needed to degrade crystalline cellulose; no other enzyme systems are known to substitute for these effects. Koenigs [55] was the first person to suggest that brown-rot fungi oxidize cellulose and that they are more powerful producers of H_2_O_2_ than white-rot fungi. He suggested that the initial attack on crystalline cellulose by brown-rot fungi is via an H_2_O_2_/Fe^2+^ system [55, 58].

The eventual involvement of H_2_O_2_ in the degradation of cellulose by brown-rot fungi has been studied in great detail by Highley [58, 59]. Initially, he determined that H_2_O_2_ might be involved in cellulose degradation by *Poria placenta*, since a decrease in cellulose was the result of the addition of -OH and H_2_O_2_ quenching agents. Additionally, he found that only one of the six brown-rot fungi studied produced significant amounts of extracellular H_2_O_2_, while several of the white-rot fungi produce extracellular H_2_O_2_. Localization of H_2_O_2_ during degradation of hemlock wood by two different fungi, the brown-rot fungus *Poria placenta* and the white-rot fungus *Coriolus versicolor* with diaminobenzidine cytochemically, the role of H_2_O_2_ in wood degradation was finally revealed [58]. A recent study of the brown-rot fungus *P. placenta* demonstrated that compounds affecting the H_2_O_2_/-OH system did not affect the degradation of wood. Thus, these results strongly suggest the involvement of extracellularly produced H_2_O_2_ in cellulose depolymerization by brown-rot fungi [55, 57–59].

### Radiation induced depolymerization

Radiation processing is a very convenient tool for imparting desirable effects in polymeric materials, and it has been an area of much interest in the last few decades. Radiation processing has been established as a commercially successful technology for the modification of a variety of synthetic polymeric materials for a variety of applications such as crosslinking of wire and cable, production of heat shrinkable materials, modification of rubber tires, and production of foamed materials. However, radiation processing of natural polymers has received much less attention because most natural polymers undergo chain sicssioning when exposed to high energy radiation making it difficult to process natural polymers in various forms and sizes [14, 19, 60]. High energy radiation techniques including gamma irradiation can be effectively used for reducing the molecular weight of polysaccharide polymers such as cellulose and alginate [61]. Conventionally synthesized low molecular weight oligosaccharides are being explored as plant growth promoters; however, until recently, the effect of radiation degradation of polysaccharide polymers was not clearly studied. The areas of applications of natural polymers being explored include health care applications and agricultural applications wherein it has been observed by a number of researchers that some low molecular natural polymers, particularly polysaccharides such as chitin/chitosan or alginates, show very interesting properties. Many natural polymers have an extremely high affinity for toxic metal ions and dyes, which makes natural polymers useful in environmental conservation due to their molecular structure [14, 19, 62].

Several related studies have been carried out in recent years on polysaccharides and their derivatives to attempt radiation-induced crosslinking in cellulose, starch, and chitin/chitosan water-soluble derivatives under various experimental conditions. Polysaccharide water soluble derivatives such as CMC, carboxymethylstarch, carboxymethylchitin, and carboxymethylchitosan are readily crosslinked when irradiated in a highly concentrated aqueous solution in paste-like state [14, 19]. Natural polymers are difficult to process and degrade when exposed to high energy radiation. Thus, radiation processing of natural polymers largely remains unexplored and industrial applications have been difficult to achieve.

The radiation technology for processing of synthetic polymers can be attributed to ease of process ability in various shapes and sizes, and most of these polymers undergo crosslinking reactions on exposure to radiation. In the case of hydrogels, which have emerged as important biomaterials as they possess excellent biocompatibility after ionizing radiation processes [18, 63]. Hydrogels are three dimensional crosslinked network structures that are produced by simultaneous polymerization and crosslinking of suitable monomers or by crosslinking of linear polymers. Ionizing radiation possesses the unique ability to initiate polymerization and crosslinking reactions without the need to add toxic chemicals, because the conventional crosslinking involves the use of toxic additives to bring about polymerization or crosslinking and thus is not suitable for biocompatible hydrogels [18, 64]. Therefore radiation processing is emerging as an excellent tool to produce hydrogels for a variety of medical applications.

### E-beam irradiated CM

**Fig. 10:**
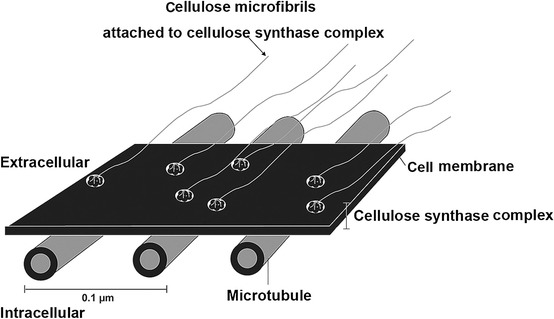
Schematic drawings of extracellular cellulose microfibrils attached to the intracellular microtubule [confirmed permission from reference journal 11]

To evaluate the effect and potential of E-beam irradiated CM, we exposed sea squirt derived CM from *Styela* clava, a non-native tunicate, to a 1–2 MeV electron beam. Cellulose is a carbohydrate polymer composed of carbon, hydrogen and β-glucose, CCP was lost after EBI, and thus the thin and delicate cellulose fibrils detached from each other by moving the cellulose synthase complex (Fig. 10) [11]. The potential of the cellulose polymer as a thin um medical membrane for guided bone regeneration by E-beam irradiated depolymerization has been suggested [11].

In our previous in vitro results, C-O bonding was increased more than C-C bonding, and several related results showed depolymerization of cellulose microfibrils composed of microtubules on high-resolution C1s and O1s ESCA spectrum (Fig. 6). In ESCA analysis, the normal spectra of C1s bindings are shown as hydrocarbon (C-C, C-H) bonding at the 284.9 eV peak, hydroxyl carbon (C-O-H) bonding at the 286.7 eV peak, ether carbon (O-C-O) bonding at the 288.4 eV peak, and ester carbon (O-C = O) bondings at the 289.6 eV peak. In the high-resolution of O1s ESCA spectrum, hydroxyl oxygen (−C-O-H), ether oxygen (−C-O-C-) and ester oxygen (−COO) bindings were differently observed [65, 66]. The peak value of 38 cps (counts per second) in the non-E-beam irradiated CM was abruptly increased to 240–250 cps after EBI, indicating a fast increase in C-O binding in surface analysis after EBI. The acceleration of oxygenation by structural changes from C-C bindings to C-O bindings on the CM surface is an important effect of depolymerization of E-beam irradiated CM [11]. This chemical shifting also can be regarded as similar to the natural reaction of H_2_O_2_ production in brown-rot fungi [55].

CM with increased C-O binding activates hydration with the surrounding water composition. The resultant hydrophilic C-OH groups have strong bioactive and hydrolysis effects. This phenomenon was confirmed using SEM (Fig. 7), which showed the many scissioning effects of C-C bindings in cellulose microfibrils by EBI with high hydrophilicity on the inner side of CM.

### Suggested E-beam effects to CM

From our small findings and related few literature review, we can suggest the possible effects of E-beam to CM as below.

1. E-beam treatment process involves accelerating a beam of electrons to near the speed of light and, by utilizing an oscillating magnetic field, sweeping the electrons back and forth across the CM.
2. Treatment of CM with an E-beam can modify physical, chemical, molecular and biological properties, so it can be studied continuously to improve its usefulness and to enhance value.
3. CCP is lost after EBI, and so the strong crosslinking binding of each cellulose fibril was broken after in vitro analysis.
4. As the dose of the E-beam increases, the inner and outer surface morphologies of CM achieve similar characteristics. Scissioning of long cellulose carbohydrate polymers can be observed in SEM findings; it is suggested that some kinds of unknown reaction of EBI must occur.
5. Depolymerizatoin processes also occur, so EBI can be used to waste some degraded cellulose fibrils. It is known that some portions of the lignin structure in plants can change their own structures.
6. E-beam irradiated CM display more hydrophilic tendencies in the ESCA because the scissioning processes in the C-O bonding are much greater than those in C-C bonding.
7. The tensile strength of CM can be changed after EBI because polysaccharide crosslinking causes a loss of resistance to compression, and uniform turgor pressure to keep the direction of the CM elongated after EBI.
8. Arrangements of microtubules can be changed indirectly, so cellulose and enzyme complexes are able to migrate in the plasma membrane by EBI.

## Conclusions

The application of nanotechnology in biomaterials engineering is one of the fastest growing areas in tissue engineering [8, 9]. Radiation has been shown to be a useful tool for arranging atoms and ions with electron beams. Radiation effects from ionizing radiation can originate either from a radioactive source or from highly accelerated electrons [9–11]. Recent advances in the understanding of EBI have resulted in new therapeutic strategies designed to improve the crosslinking of polymer-based products, the degradation of recycled materials, and the sterilization of medical and pharmaceutical goods. In this article, we focused on CM.

### Availability of supporting data

Recent advances in the understanding of EBI have resulted in new therapeutic strategies designed to improve the crosslinking of polymer-based products, the degradation of recycled materials, and the sterilization of medical and pharmaceutical goods. In this article, we review clinical applications of CM, cellulose binding domains, cellulose crosslinking proteins, conventional hydrolysis of cellulose, and depolymerization with radiation and focus our experiences with depolymerization of E-beam irradiated CM.
